# Oxford War Primers

**Published:** 1916-07

**Authors:** 


**Oxford War Primers.** London: Henry Frowde & Hodder and Stoughton. 1915.

A very useful purpose is subserved by the timely publication of these handy primers, any two of which can be readily carried in the officer's uniform pocket.

*Wounds in War*, by Lieut.-Col. D'Arcy Power (pp. 108), the first we turn to, reminds us on the first page that the effect of the present war has been to throw us back to the time before Lister, when most wounds suppurated, for the wounds to be treated are already for the most part deeply infected before there is any possibility of treating them. "Some few things have had to be unlearnt. We know, for instance, why our forefathers made such large and deep incisions, why they amputated so freely, and why they preferred simple circular amputations to the more elaborate, and, as it seemed to us, the better method of cutting flaps. We know, too, why they valued a sound knowledge of the gross anatomy of arteries and nerves, laying very little stress upon the smaller details, whilst their preference for poultices and wet dressings has been justified." Such a paragraph as we quote is of comfort, not for itself, but for what it implies, viz. that we must be prepared to revise our ideas in dealing with the wounded on the modern battle-field, and throughout these text-books we find that the experiences of those who in their several departments have more than justified the enterprise of the publishers. Much of the space of the particular volume we now have before us is devoted to practical if elementary surgical points, such as the ligation of various arteries which, while not usually coming under treatment in civil practice, may require immediate ligation in the trenches. But the sections on the preparation of vaccines, their uses, and the employment of sensitised vaccines, show that the work is thoroughly modern.

*Medical Hints*, by Colonel J. Edward Squire, is full of useful notes, though why tonsillitis should be included in this volume it is not easy to guess. We see that the author suggests atropine for the bronchitis of chlorine gassing.

*Cerebro-Spinal Fever*, by Thomas J. Horder, epitomises current knowledge of the disease. The author rightly emphasises the importance of following no rule of thumb method of administering Flexner's serum, but of pushing it in the super-acute types.

*Nerve Injuries and Shock*, by Captain Wilfred Harris, is approached mainly from the physician's standpoint. It contains a very useful chapter on nervous shock.

*Wounds of the Thorax in War*, by J. Keogh Murphy, *Injuries to Joints*, by Major Robert Jones, and *Abdominal Injuries*, by Professor Rutherford Morison and Lieut.-Col. W. G. Richardson, are in each case excellent.

*Gunshot Injuries of Bones*, by Captain E. W. Hey Groves, is one of the most original of the series. The author's extensive contributions to the furtherance of methods of fracture treatment have proved of great practical value in the treatment of bone injuries in war. Many useful suggestions will be found in the section on "Bone Grafts."

*Surgery of the Head*, by Major Bathe Rawling, and *Injuries of the Eyes, Nose, Throat and Ears*, by Major Andrew Maitland Ramsay, Major Dundas Grant, Captain Lawson Whale and Captain C. Ernest West, complete the series.

The latter volume contains short notes of some very interesting illustrative cases, and among them a case showing the value of tracheoscopy and bronchoscopy in diagnosing the exact nature of certain chest injuries.

While the standard of excellence reached in some volumes is perhaps higher than others, we have felt it unnecessary and even difficult to indicate this difference in any way, for every volume is of very great value. The type is clear, the illustrations are all good, and the copious index to each volume adds greatly to the facility of ready reference to any point on which information is desired.

